# Effect of the G375C and G346E Achondroplasia Mutations on FGFR3 Activation

**DOI:** 10.1371/journal.pone.0034808

**Published:** 2012-04-18

**Authors:** Lijuan He, Christopher Serrano, Nitish Niphadkar, Nadia Shobnam, Kalina Hristova

**Affiliations:** Department of Materials Science and Engineering, Johns Hopkins University, Baltimore, Maryland, United States of America; Institut Jacques Monod, France

## Abstract

Two mutations in FGFR3, G380R and G375C are known to cause achondroplasia, the most common form of human dwarfism. The G380R mutation accounts for 98% of the achondroplasia cases, and thus has been studied extensively. Here we study the effect of the G375C mutation on the phosphorylation and the cross-linking propensity of full-length FGFR3 in HEK 293 cells, and we compare the results to previously published results for the G380R mutant. We observe identical behavior of the two achondroplasia mutants in these experiments, a finding which supports a direct link between the severity of dwarfism phenotypes and the level and mechanism of FGFR3 over-activation. The mutations do not increase the cross-linking propensity of FGFR3, contrary to previous expectations that the achondroplasia mutations stabilize the FGFR3 dimers. Instead, the phosphorylation efficiency within un-liganded FGFR3 dimers is increased, and this increase is likely the underlying cause for pathogenesis in achondroplasia. We further investigate the G346E mutation, which has been reported to cause achondroplasia in one case. We find that this mutation does not increase FGFR3 phosphorylation and decreases FGFR3 cross-linking propensity, a finding which raises questions whether this mutation is indeed a genetic cause for human dwarfism.

## Introduction

Receptor tyrosine kinase (RTK)-mediated signaling regulates vital cellular processes such as cell growth, differentiation, and motility [1]–[5]. The activation of RTKs is initiated with the phosphorylation of specific tyrosines in their intracellular kinase domains [6], [7]. For phosphorylation to occur, the kinase domains of two RTKs need to be in close proximity, and the orientation and the positioning of the kinases with respect to each other need to be tightly controlled to ensure optimal enzymatic activity. The close approach of the kinase domains is regulated by the propensities of RTKs to form dimers in the plasma membrane, and is modulated by ligand binding to RTK extracellular domains [8]. Specific receptor-receptor and receptor-ligand interactions in the dimer impose structural constraints and ensure the correct signaling-competent orientation of the kinase domains [9].

Disregulation of the processes that control RTK activation leads to pathologies [10]–[13]. Many single amino acid pathogenic RTK mutations have been identified, and the molecular mechanism behind these pathologies has been investigated [14]–[21]. While the progress in this field has been impressive, still questions remain as to how RTK mutations cause disease. For instance, it is not yet known why one group of mutations in an RTK causes the same phenotype, while another group of very similar mutations in the same RTK causes a distinctly different phenotype. For instance, three different mutations in FGFR3, G346E, G375C, and G380R have been linked to achondroplasia (ACH), the most common form of human dwarfism, while several other mutations in the same receptor (R240C, R248C, S249C, 370C, S371C, Y373C, and K650E) cause the much more severe and lethal thanatophoric dysplasia type 1 (TD1) [10], [11], [22].

Thus far, the effect of FGFR3 mutations linked to phenotypes of different severities have been compared and rank-ordered, and mutations associated with more severe phenotypes have been shown to have a more profound effect on FGFR3 signaling. For instance, mutations associated with TD1 have been shown to activate FGFR3 to higher extent than mutations causing ACH [23]. However, the effect of mutations causing the same phenotype has not been compared in order to determine if they activate the receptors to the same, or to variable, extents, and whether the mechanism of over-activation leading to a particular phenotype is always the same.

To start addressing this question, here we investigate the effect of the G375C mutation, linked to ACH, on FGFR3 phosphorylation and cross-linking, and we compare the results to previously published results for the G380R ACH mutant. The comparison is carried out over a wide range of ligand concentrations, in order to gain insight into the molecular mechanism that underlies the pathology. We observe that the G375C mutation increases FGFR3 phosphorylation in the absence of ligands and at low, but not at high, ligand concentrations, similarly to the G380R mutation. The dimerization propensity of FGFR3 is not affected by the G375C mutation, as inferred from cross-linking experiments. Our results demonstrate that the two achondroplasia mutations, G375C and G380, have the same effect on FGFR3 activity, supporting the idea that identical degrees and mechanisms of over-activation lead to identical phenotypes.

In addition, here we investigate a third FGFR3 mutation, G346E. While this mutation is often mentioned in the literature as a genetic cause for ACH [12], [24]–[29], there is a single report of a connection between this mutation and ACH [30]. The effect of this mutation on FGFR3 activation has not been studied thus far. Here we perform biochemical characterization of the G346E mutant and we show that the G346E mutation does not increase FGFR3 phosphorylation, a finding which raises doubts whether this mutation is indeed a genetic cause for human dwarfism.

## Materials and Methods

### Plasmids

The FGFR3/WT plasmid was a generous gift from D. J. Donoghue, University of California, San Diego. The G375C and G346E mutations were created using the QuickChange® XL site-directed mutagenesis kit (Stratagene, CA). All the plasmids were in the pcDNA 3.1(+) vector. Successful incorporation of the mutations was confirmed by sequencing provided by Genewiz (Germantown, MD).

### Western Blots

Human embryonic kidney cells (HEK 293 from **ATCC**) were transfected with plasmids encoding FGFR3/WT, FGFR3/G375C and FGFR3/G346E using FuGENE HD (Roche Applied Science) according to the manufacturer's protocol. Cells were cultured in normal medium for 24 h following transfection, and were then starved in serum-free medium for 24 h as previously described [17], [31]–[34]. The effect of the ligand was monitored by incubating the cells in medium supplemented with **fgf1** (Millipore, MA). Different concentrations of **fgf1**, ranging from 50 to 2500 ng/ml, were added to the serum-free medium. After incubating for 10 min with ligand, cells were lysed as described [17], [31]–[33], and analyzed using Western blotting. FGFR3 expression and phosphorylation were probed with anti-N-FGFR3 (H-100) antibodies (sc-9007, Santa Cruz Biotechnology) or anti-P-FGFR antibodies (anti-Tyr-653/Tyr-654, Cell Signaling Technology), respectively, followed by anti-rabbit HRP-conjugated antibodies (Promega). The Western blot films were scanned and processed with ImageQuant TL. At least three sets of experiments were performed to determine the averages and the standard deviations. The loading of the gels was adjusted such that all the band intensities were within the so-called linear range, such that the staining intensities were proportional to the protein concentration [17].

### Cross-linking

Dimeric receptors were cross-linked with a membrane-impermeable linker (BS^3^, bis(sulfosuccinimidyl) suberate, Pierce). Twenty four hours after transfection, cells were incubated with 2 mM cross-linker for 30 min at room temperature and then quenched in 20 mM Tris-HCl for 15 min. After a rinse with ice-cold PBS, the cells were lysed, and the receptors were detected using Western blotting. The cross-linked fraction was calculated as *S_D_/S* = *S_D_*/(*S_M_*+*S_D_*), where *S_D_* is the intensity of the cross-linked band, and *S_M_* is the intensity of the monomeric band [32].

### Statistical Analysis

All experiments were repeated at least three times to determine the averages and the standard deviations. Student t-test was used to compare the measurements to a null hypothesis for statistical significance, as previously described [33], [34]. The p-value cutoff for significance was determined as 0.017 using the Bonferroni correction for multiple comparisons.

## Results

### Effect of the G346E and G375C mutations on FGFR3 expression pattern in HEK 293 cells

**Figure 1:**
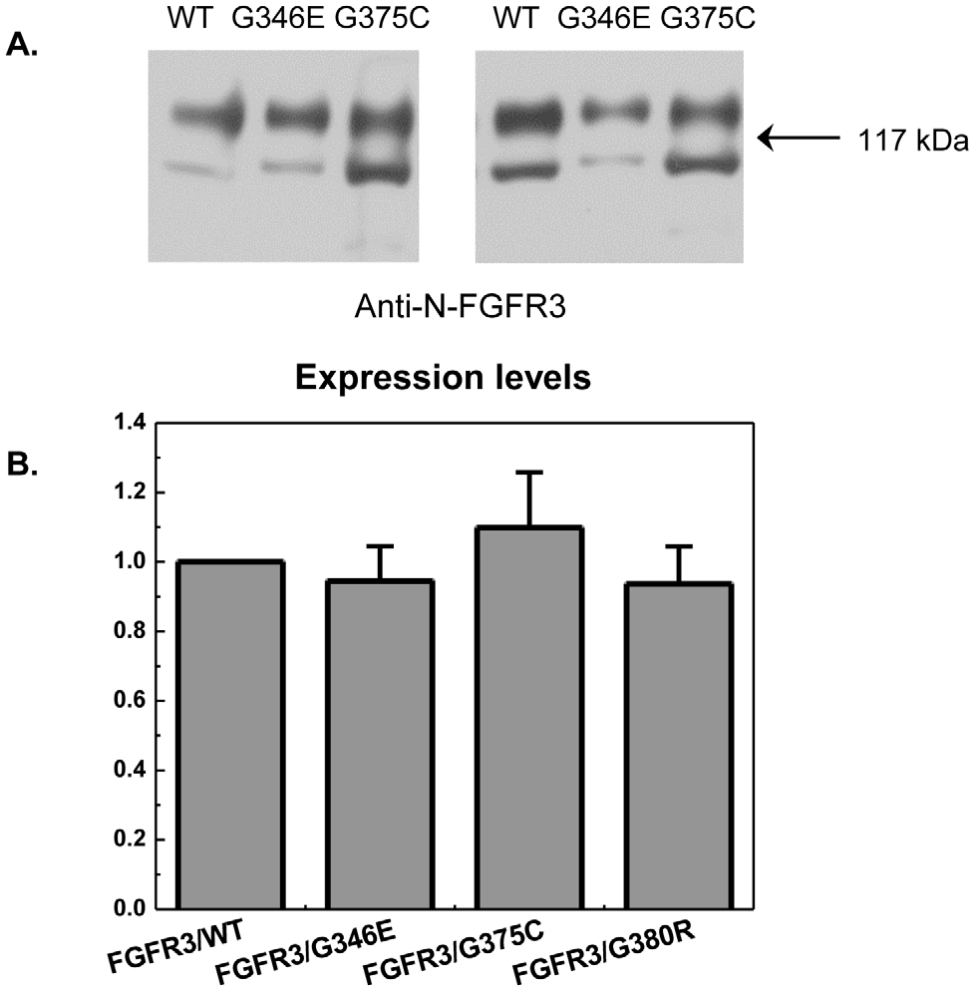
FGFR3 expression patterns in HEK 293 cells. Cells were transfected with 2 µg/well of DNA encoding FGFR3/WT, FGFR3/G346E, or FGFR3/G375C. 24 hours after transfection, cells were starved in serum-free medium, followed by cell lysis. The cell lysates were subjected to Western blotting, and FGFR expression was probed using anti-N-FGFR3 antibodies. **A.** Two representative blots, showing the expression of FGFR3/WT, FGFR3/G346E and FGFR3/G375C. **B.** Quantification of the expression of the 130 kDa mature FGFR3, based on results from ten independent experiments. There are no statistical differences between the expressions of the wild-type and the three mutants (p>0.017, see text). Data for the G380R mutant are from [35].

HEK 293 cells were transfected with 2 µg of DNA encoding FGFR3/WT, FGFR3/G346E or FGFR3/G375C using Fugene HD. 24 hours after transfection, cells were starved in serum-free medium and lysed. The cell lysates were subjected to Western blotting. The expression of wild type FGFR3 and the two mutants was probed using anti-N-FGFR3 antibodies. Ten independent experiments were performed, with two representative results shown in Figure 1A. We observe two bands reactive to the anti-N-FGFR3 antibodies in all cases. As discussed in previous publications [33]–[35], the top band corresponds to the fully glycosylated mature 130-kDa FGFR3 form, which is located predominantly in the plasma membrane. The lower band corresponds to the 120-kDa FGFR3 form, which is sensitive to endo-β-*N*-acetylglucosaminidase H and is found in the endoplasmic reticulum/cis-Golgi. In Figure 1, we see that the transient expression levels of the two mutants and the wild-type are somewhat different in each experiment under identical transfection conditions, which can be expected for transient transfection. The variation of the expression of the 120 kDa form is more pronounced than the variation in the 130 kDa form expression. To quantify the expression of the 130 kDa forms of the wild-type and the two mutants, we measured the intensities of the 130 kDa bands using Image Quant, normalized all the intensities with respect to the wild-type intensity, and averaged the results from the 10 independent experiments. The results, shown in Figure 1B, demonstrate that neither of the two mutations affects the expression of mature FGFR3 in HEK 293 cells in a statistically significant way (p values: 0.44 and 0.40 for the G346E and the G375C mutations, respectively). In Figure 1B we also show published results for the G380R mutant (p value: 0.33). These results demonstrate that the G380R mutation does not affect the expression of mature FGFR3, either [35].

Close inspection of the FGFR3/G375C Western blot results in Figure 1 reveals a weak, but always visible, third band reactive to anti-N-FGFR3 antibodies. Based on molecular weight, this band should correspond to the unglycosylated 98-kDa receptor. This finding suggests that a small portion of FGFR3/G375C fails to be modified/glycosylated and is likely trapped inside the cells.

### Response of the G346E and G375C mutants to the ligand fgf1

Next we characterized the response of the two FGFR3 mutants to different concentrations of **fgf1**. HEK 293 cells were transfected with plasmids encoding FGFR3/WT, FGFR3/G346E or FGFR3/G375C. Different concentrations of **fgf1**, ranging from 50 to 2500 ng/ml, were added to the serum-free medium. After incubation for 10 min with ligand, cells were lysed and analyzed using Western blotting. The receptors were stained with anti-N-FGFR3 (H-100) antibodies or anti-P-FGFR antibodies. The anti-P-FGFR antibodies used were anti-phospho-Tyr-653/Tyr-654 antibodies (Cell Signaling), generated to recognize phosphorylated Tyr-653 and Tyr-654 in FGFR1. Since the amino acid sequences of FGFR1 and FGFR3 in the vicinity of these two tyrosines are the same, it also specifically recognizes the analogous Tyr-647 and Tyr-648 in FGFR3 [35].

**Figure 2:**
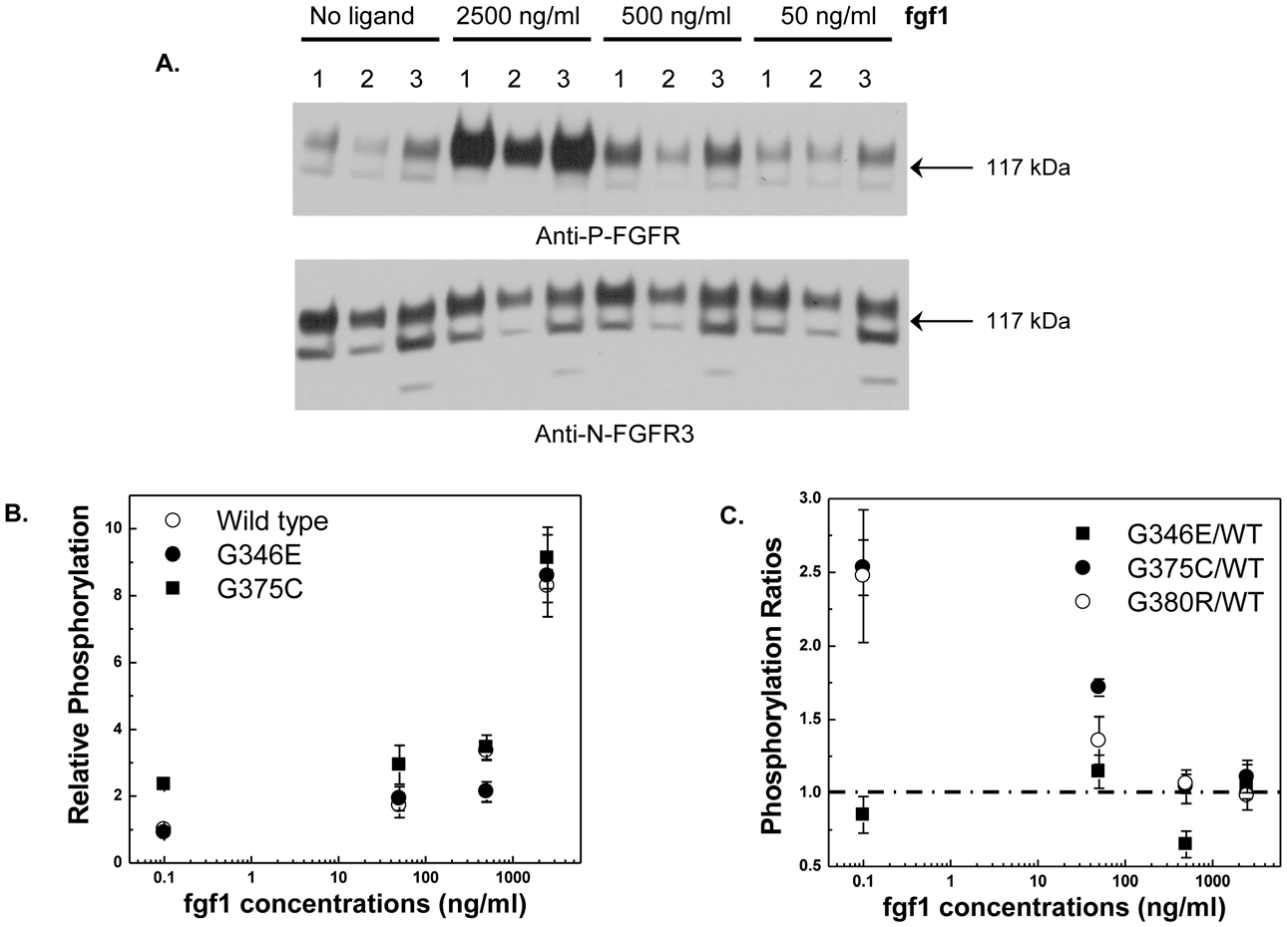
Effect of the achondroplasia mutations on FGFR3 phosphorylation. HEK 293 cells were transfected with plasmids encoding FGFR3/WT, FGFR3/G346E and FGFR3/G375C. Different concentrations of **fgf1**, ranging from 50 to 2500 ng/ml, were added to the serum-free medium. After incubating for 10 min with ligand, cells were lysed and analyzed using Western blotting. **A.** A typical Western blot result. FGFR3 was detected with anti-N-FGFR3 (H-100) antibodies (bottom) or anti-P-FGFR antibodies (top). Lanes 1: FGFR3/WT, Lanes 2: FGFR3/G346E, Lanes 3: FGFR3/G375C. **B.** The relative phosphorylation levels of the receptors were calculated by dividing the anti-P-FGFR staining intensities by the anti-N-FGFR3 staining intensities. The phosphorylation levels increase with ligand concentration. C. The phosphorylation levels of FGFR3/G375C, FGFR3/G346E and FGFR3/G380R were divided by that of FGFR3/WT. The G380R data are from [35].

Three independent experiments were performed while varying the ligand concentration. One representative film is shown in Figure 2A. Only the mature FGFR3 130 kDa form, expressed predominantly on the plasma membrane, responds to the ligand, while the lower molecular weight FGFR3 located in the endoplasmic reticulum does not show increased phosphorylation upon ligand titration. The intensities of the 130 kDa anti-N-FGFR3 and anti-P-FGFR bands were quantified, and their ratio yielded the relative phosphorylation level of the receptors. In each individual experiment, the relative phosphorylation level of wild type FGFR3 in the absence of ligand was assigned the value of one, and all the phosphorylation levels were scaled accordingly and plotted in Figure 2B. We see that in the absence of ligand, the phosphorylation of the G375C mutant is higher than the phosphorylation of the wild-type and the G346E mutant. All phosphorylation levels increase with ligand concentration, while the difference between the mutants and the wild-type decreases.

In Figure 2C, we plot the ratio of the mutant and wild-type phosphorylation levels, as a function of ligand concentration, while also including previously published data for the G380R mutant [35]. We see that in the absence of ligand, the phosphorylation of both the FGFR3/G375C and the FGFR3/G380R mutants is about 2.5 times higher than that of the wild type. This difference is statistically significant (p-values for the G375C/WT and G380R/WT comparisons are 0.015 and 0.001 respectively). As more ligand is added, the difference between these mutants and the wild type gradually decreases. On the other hand, the FGFR3/G346E mutant does not show any increase in phosphorylation, as compared to wild type FGFR3 (p = 0.35). When the concentration of ligand is above 500 ng/ml, there is no statistical difference between the phosphorylation of FGFR3/G346E, FGFR3/G375C, FGFR3/G380R and FGFR3/WT (p values: 0.82, 0.46 and 0.45 for the G346E/WT, G375C/WT and G380R/WT comparisons, respectively).

### Effect of the G346E and G375C mutations on FGFR3 cross-linking

**Figure 3:**
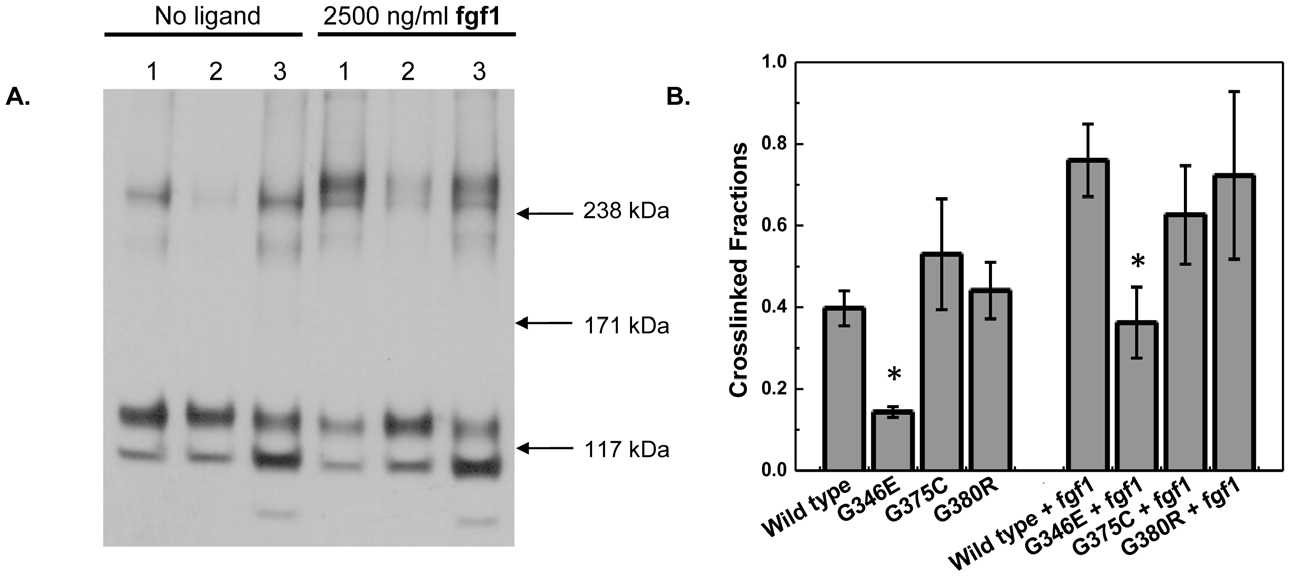
Effect of the achondroplasia mutations on FGFR3 cross-linking. HEK 293 cells were transfected with 2 µg DNA per well. 24 hours after transfection, cells were starved in serum-free medium for 24 hours, followed by ligand addition, BS^3^ crosslinking, and lysis. **A.** A typical Western blot result. Lane 1: FGFR3/WT, Lane 2: FGFR3/G346E, Lane 3: FGFR3/G375C. **B.** Cross-linked fractions, calculated as S_D_/S = S_D_/(S_M_+S_D_), where S_D_ is the intensity of the cross-linked band, and S_M_ is the intensity of the monomeric band. The stars indicate statistical significance (p<0.01, see text).

Next we used chemical cross-linking to gain insight into the effect of the mutations on FGFR3 dimerization. HEK 293 cells were transfected with plasmids encoding FGFR3/WT, FGFR3/G346E or FGFR3/G375C. Cells were starved for 24 hours, and stimulated with high concentration of **fgf1**. Cells that were not stimulated with ligand were used as controls. BS3, a membrane impermeable linker, was used to covalently link the FGFR3 dimers on the plasma membrane. Cells were lysed and the lysates were analyzed using Western blotting. A typical Western blot film is shown in Figure 3A. The cross-linked fraction was calculated as S_D_/S = S_D_/(S_M_+S_D_), where S_D_ is the intensity of the cross-linked band, and S_M_ is the intensity of the monomeric band. The results are shown in Figure 3B, together with previously published results for the G380R mutant [35]. Figure 3B demonstrates that in the absence of ligand, the cross-linked fractions of FGFR3/G375C, FGFR3/G380R and FGFR3/WT are very similar (p-values: 0.24 and 0.20 for the G375C/WT and the G380R/WT comparison, respectively), whereas the FGFR3/G346E cross-linked fraction is lower than wild type (p = 0.0009). Upon the addition of **fgf1**, all three receptors show a moderate increase (∼50% to 100%) in cross-linked fraction. In the presence of ligand, the cross-linked fraction for wild type FGFR3 and the G375C and G380R mutants remain similar (p-values: 0.2 and 0.74 for the G375C/WT and the G380R/WT comparison, respectively), and the G346E cross-linked fraction remains lower (p = 0.005). The results indicate that none of the three ACH mutations increases FGFR3 crosslinking, and thus the dimerization propensity of FGFR3. Therefore, the increase in phosphorylation of the G375C or G380R mutants, shown in Figure 2, is likely not due to increased receptor dimerization.

## Discussion

### The physical basis behind FGFR3 disregulated signaling in ACH

Achondroplasia is a bone-growth disorder with phenotypic features which include short arms and legs, and a large head with prominent forehead [36], [37]. It is often caused by a G380R mutation in the transmembrane domain of FGFR3, a receptor which mediates pro-differentiation signals in chondrocytes [38]. The G380R mutation has been shown to increase FGFR3 phosphorylation and thus shorten the proliferative phase in chondrocyte development, but the cause for the increased receptor phosphorylation is under debate. Webster and Donoghue proposed that the G380R mutation enhances FGFR3 dimerization, likely due to hydrogen bonding of the mutant R380 to the neighboring receptor in the dimer [39]. However, He et.al showed that while FGFR3 phosphorylation is increased due to the mutation, FGFR3 cross-linking and dimerization propensities are not affected by the mutation [35]. Instead, the cross-phosphorylation efficiencies within the unliganded FGFR3 dimers are increased [35]. Based on these results, and on a molecular model of FGFR3 TM domain created with the program CHI [16], [40], it has been proposed that the G380R mutation introduces a structural change in the unliganded FGFR3 dimers, thus altering their phosphorylation efficiencies and ultimately increasing FGFR3 phosphorylation in the absence of ligand.

The G380R mutation accounts for about 98% of the ACH cases. The other 2% are attributed to the G375C mutation in FGFR3 [11]. Since this is a mutation to cysteine, it can be expected to increase the stability of FGFR3 dimers due to the formation of a disulfide bond. Here we demonstrate, however, that the mutation does not increase FGFR3 cross-linking, and thus we observe no evidence for dimer stabilization due to the formation of disulfide bonds. Similarly to the G380R mutation, we see that the phosphorylation of the G375C mutant is increased at low ligand concentrations, but the mutation does not affect FGFR3 phosphorylation at high ligand concentrations. Thus, the G375C mutation must increase the receptor phosphorylation probability within unliganded FGFR3 dimers. In other words, it is easier to phosphorylate Y647 and Y648 within the mutant unliganded dimer than within the wild-type unliganded dimer. The most likely reason for this difference in phosphorylation is a difference in the structure of the unliganded dimer. We thus propose that the G375C achondroplasia mutation induces a structural change in the unliganded dimer, similarly to the G380R mutation.

A published model of the wild-type dimer structure, created with the program CHI, predicts that Glycine in position 375 does not face the dimer interface and is exposed to lipids [41]. Substitution of Glycine with cysteine at position 375 shows that the two Cysteines in the dimer are far apart and cannot be expected to form a disulfide bond [41]. Our data are consistent with this molecular model because we do not observe an increase in the number of dimers due to the G375C mutation, as inferred from cross-linking experiments. Our data also suggests, however, that the structure of the FGFR3 TM domain dimer is perturbed due to the mutation, and this perturbation of unknown nature affects the activity of the kinase domain. The G380R mutation has been proposed to induce a rotation in the TM dimer interface [35]. Since the phosphorylation increase is identical due to these two achondroplasia mutations, it can be speculated that a similar structural change may be taking place in the presence of the G375C mutation.

Our work is consistent with previous studies which have reported increased phosphorylation of the G375C mutant, as compared to wild-type [19]. Furthermore, the lack of disulfide bond-mediated stabilization that we observe here is consistent with a previous FRET-based study of the dimerization of isolated FGFR3 TM domains in lipid bilayers [41]. On the other hand, our cross-linking results for the FGFR3/G375C mutant are different from previous work done by Adar et.al, who observed increased FGFR3 cross-linking due to the G375C mutation [19]. This difference may be due to the experimental methods used: for instance, Adar et al assayed cross-linking after immunoprecipitation, while here we do not use immunoprecipitation.

Here we observe that FGFR3 phosphorylation in the absence of ligand increases 2.5 times due to the G375C and G380R mutations. The effect is greatest in the absence of ligand and the mutation-induced difference in phosphorylation decreases as more ligand is added. Thus, a question arises if an effect of this magnitude can be responsible for a pathological phenotype. It is possible that the effect is amplified due to defects in mutant FGFR downregulation. It has been shown that the downregulation of FGFR3 mutants linked to skeletal dysplasias is compromised, and this effect prolongs the time that they signal [10], [42]–[44]. Importantly, the relative magnitudes of these processing defects have been shown to be proportional to the phosphorylation of the mutants: the higher the phosphorylation, the longer the lifetime of the active FGFR3 dimers in the cell [10], [43], [45]. Such downregulation defects should, ultimately, increase the concentrations of the mutants in the cell, as compared to the wild-type. We did not see statistically significant differences in receptor concentrations in our experiments, which may be due to the fact that in this study all receptors are overexpressed. We note, however, that we observed an un-glycosylated band for the FGFR3/G375C mutant, which is not observed for wild-type FGFR3 or other FGFR3 mutants. Thus, it is possible that the FGFR3/G375C mutation interferes with the maturation and trafficking of the receptors. The intensity of the unglycosylated band observed here, however, is very weak, and thus this effect appears very modest.

### G346E is not a gain-of-function mutation

The G346E mutation in FGFR3 has been reported to cause achondroplasia [30], and is often cited in the literature as a genetic cause for the disorder [12], [24]–[29]. However, there are no biochemical studies of the effect of this mutation on FGFR3 phosphorylation in the literature. Here we studied the effect of the G346E mutation on FGFR3 signaling in HEK 293 cells, and we showed that the mutation does not increase FGFR3 phosphorylation. This conclusion contradicts the expectation that G346E is a gain-of-function mutation. Since there is a single report of a link between this mutation and ACH [30], our findings raise doubts about its validity. While the observed decrease in cross-linking for the G346E mutant warrants further investigation, it is difficult to envision how the decrease in cross-linking may be related to the disorder. The activation data demonstrate that the G346E mutation is not a gain-of-function mutation and thus this mutation is likely not a genetic cause for ACH.

### FGFR3 phosphorylation levels determine the severity of dwarfism phenotypes

It has been suggested that there is a correlation between the level of FGFR3 phosphorylation and the severity of the disturbance in endochondrial ossification in human skeletal dysplasias. Ornitz and colleagues were the first to explore if such a correlation exists [23]. They compared the ligand-independent phosphorylation of the R248C and K650E mutants, associated with thantaophoric dysplasia type 1 (TD1), to the phosphorylation of the G380R mutant linked to ACH. TD1 is characterized by severe shortening of the limbs with curved femurs, large head, and a narrow thorax with small ribs [11], [22]. It is always lethal in the neonatal period, and is thus much more severe than ACH. In the experiments of Ornitz and colleagues, the mutations responsible for TD1 were more activating than the ACH mutation [23]. Thus, these experiments provided a biochemical explanation of the severity of the two phenotypes.

A study by Bellus at al [46] compared the ligand-independent phosphorylation of FGFR3 mutants implicated in (i) thanatophoric dysplasia type 2 (TD2), (ii) severe achondroplasia with developmental delay and acanthosis nigricans (SADDAN), and (iii) hypochondroplasia (HCH). The clinical features of TD2 are similar to the clinical features of TD1, but the femurs in TD2 are straight. SADDAN patients have very short stature with very short limbs, and exhibit developmental delay and intellectual disability. The features of HCH, which occurs in 1 in 15,000 to 40,000 newborns, are milder than ACH. The work of Bellus compared the ligand-independent activity of FGFR3 carrying three different mutations: the K650E mutation linked to TDII, the K650M mutation causing SADDAN, and the K650N and K650Q mutations associated with hypochondroplasia. They found that the phosphorylation of the TD2 and SADDAN mutants was much higher than that of the HCH mutants, which in turn was higher than the phosphorylation of the wild-type [46].

In a third study, Adar and colleagues compared the effects of the G370C and G371C mutations linked to TD1, to the effect of the ACH G375C mutation [19]. The G370C and S371C mutants produced higher levels of constitutive activation of MAPK in L8 cells and c-*fos* transcription in RCJ cells than the G375C mutant, consistent with the fact that TD1 is much more severe than ACH.

All these data suggest that a relationship exists between the activity levels of the mutants and the severities of the associated disorders. Here we seek insight into how *different* FGFR3 mutations cause the *same* dwarfism phenotype, and we thus compare the phosphorylation of two mutants linked to the same phenotype, ACH. Unlike previous comparisons, here we compare the phosphorylation over a wide range of ligand concentration, and we perform cross-linking experiments within the native cellular membrane in order to probe the underlying cause for the elevated phosphorylation due to the mutations. We observe identical behaviors of the G380R and G375C mutants in terms of their activities and cross-linking propensities. These data are consistent with the idea that the severity of the phenotype is directly linked to the degree of RTK phosphorylation.

Yet, the phosphorylation level of a particular mutant is not the only determinant of a pathological phenotype. For instance, the A391E mutation in FGFR3 increases FGFR3 phosphorylation at low ligand concentration, similarly to the G380R and G375C ACH mutations studied here [31]. However, the A391E mutation causes a distinctly different phenotype, Crouzon Syndrome with Acanthosis Nigricans, a disorder which impacts predominantly the cranial bones and the skin [47], not the long bones. It has been shown that the A391E mutation increases FGFR3 dimerization [16], [31], [40], unlike the G380R and G375C mutations. Thus, both the level of kinase activity, and the physical-chemical and structural determinants of the kinase activity, determines a specific pathological phenotype.
